# Feel the Time. Time Perception as a Function of Interoceptive Processing

**DOI:** 10.3389/fnhum.2018.00074

**Published:** 2018-03-06

**Authors:** Daniele Di Lernia, Silvia Serino, Giovanni Pezzulo, Elisa Pedroli, Pietro Cipresso, Giuseppe Riva

**Affiliations:** ^1^Department of Psychology, Università Cattolica del Sacro Cuore, Milan, Italy; ^2^Applied Technology for Neuro-Psychology Lab, IRCCS Istituto Auxologico Italiano, Milan, Italy; ^3^Institute of Cognitive Sciences and Technologies, National Research Council, Rome, Italy

**Keywords:** interoception, interoceptive buffer, C-T fibers, time perception

## Abstract

The nature of time is rooted in our body. Constellations of impulses arising from the flesh constantly create our interoceptive perception and, in turn, the unfolding of these perceptions defines human awareness of time. This study explored the connection between time perception and interoception and proposes the Interoceptive Buffer saturation (IBs) index. IBs evaluates subjects’ ability to process salient stimuli from the body by measuring subjective distortions of interoceptive time perception, i.e., the estimated duration of tactile interoceptive stimulations. Thirty female healthy subjects were recruited through consecutive sampling and assessed for common variables related to interoceptive alterations: depressive symptoms (Beck Depression Inventory, BDI-II), eating disorders (EDI-3) risk, and anxiety levels (State Trait Anxiety Inventory, STAI). Interoceptive cardiac accuracy (IAc) was assessed as well. Subjects performed verbal time estimation of interoceptive stimuli (IBs) delivered using a specifically designed interoceptive tactile stimulator, as well as verbal time estimation of visual and auditory stimuli. Results showed that IBs index positively correlated with IAc, and negatively with EDI-3 Drive for Thinness (DT) risk subscale. Moreover, IBs index was positively predicted by IAc, and negatively predicted by DT and somatic factors of depression. Our results suggest that underestimations in interoceptive time perception are connected to different psychological conditions characterized by a diminished processing of high salience stimuli from the body. Conversely, overestimations of the duration of interoceptive stimuli appear to be function of subjects’ ability to correctly perceive their own bodily information. Evidence supported IBs index, fostering the concept of interoceptive treatments for clinical purposes.

## Introduction

Time perception is a fundamental element of human awareness. Our consciousness, our ability to perceive the world around us and, ultimately, our very sense of self are shaped upon our perception of time in loop connecting memories of the past, present sensations and expectations about the future. Yet, the way we perceive time is widely debated.

Scalar Expectancy Theory (Gibbon et al., 1984) is one of the most accepted frameworks of time perception (Church, 1984; Treisman et al., 1990). A core tenet of SET is an internal clock with a pacemaker-accumulator component. Pulses emitted by the pacemaker are stored in the accumulator, and the amount of units stored in a finite span influences sample frequency and our time perception. A high pulse rate will store more units in the accumulator, therefore leading to overestimation in subjective perception, whereas a low pulse rate will produce opposite effects. Recent developments of SET included memory and decision making components along with the pacemaker-accumulator unit, providing a more efficient neurocognitive framework for time awareness (Gibbon et al., 1984; Lui et al., 2011). Moreover, the Attentional Gate Model (Zakay and Block, 1996) introduced attention as mediator of the mode switch between the pacemaker and the accumulator. Specifically, attention can control the mode switch (Wearden and Penton-Voak, 1995; Droit-Volet and Meck, 2007; Wearden et al., 2010; Ogden et al., 2015a) in such a way that, if the switch is open, some emitted pulses can be lost therefore contracting our perception of time (Burle and Casini, 2001; Wittmann, 2009; Ogden, 2013).

The pacemaker-accumulator framework has been connected to an embodied model of time perception (Wittmann and van Wassenhove, 2009; Wittmann et al., 2010) by several authors. In this perspective, bodily states and emotions represent central elements whereas high physiological arousal (Gil and Droit-Volet, 2012; Grecucci et al., 2014; Ogden et al., 2015a; Yoo and Lee, 2015; Mioni et al., 2016) can increase the pulse frequency of the pacemaker, creating overestimation of subjective time perception (Wittmann, 2009). Numerous other authors also suggested that arousal and bodily signals are deeply connected with subjective time awareness (Droit-Volet and Gil, 2009; Gil and Droit-Volet, 2012; Pollatos et al., 2014; Ogden et al., 2015a,b), leading to the conclusion that perception of time is intimately rooted in our body.

An embodied perspective of time is also supported by various computational neuroscientific models that highlight the importance of the body and of bodily movements. Evidence from Tomassini and Morrone (2016) suggested that subjective perception of time is connected to the motor cortex whereas sensory-motor conflicts integration contributes to subjective distortion of time across different modalities. In a similar way, Orgs et al. (2013) linked the perception of time to the processing of human movements that subsides visual and motor cortical areas. Moreover, complementary evidence identified a direct link between temporal evaluation and visuo-motor representation of motor actions, highlighting the connection between time perception and body movements (Gavazzi et al., 2013) also on representative level. Perception of time appears therefore as a fundamental requirement of several functions related to the body. To this account, Buonomano and Laje (2010) proposed the concept of “population clocks” that envisions perception of time as an emerging trait of recurrently connected neural networks that encode time, and specifically motor time, in the activity patterns of a population of neurons (Buonomano, 2014). Along with previous discussed evidence, these converging data support therefore an embodied perspective of time perception in humans (Kranjec and Chatterjee, 2010; Wittmann, 2014) which integrates several sources, from emotions to body movements, to create our awareness of time.

Further support to the embodied perspective of time comes from Craig’s recent work on the “interoceptive matrix” and its relations to human time awareness. The interoceptive matrix located in the anterior insular cortex (AIC) receives afferent inputs from small diameter sensory fibers through the lamina I spinothalamocortical pathway, which carries fundamental information from all the tissues of the body (Craig, 2002, 2003, 2009; Gu et al., 2013; Gu and FitzGerald, 2014) creating interoceptive perceptions. Recent studies showed selective activation of the AIC in time perception (Rao et al., 2001; Coull, 2004; Lewis and Miall, 2006; Livesey et al., 2007) specifically within the range of sub-seconds to seconds, confirming AIC as one of the core constituents of human awareness of time.

A central component of the interoceptive matrix is the interoceptive buffer (Craig, 2009) that processes and compares interoceptive information with previous and past states of the body, in order to predict future conditions of the organism (Friston, 2009, 2010; Friston et al., 2011; Seth, 2013; Ondobaka et al., 2017). These interoceptive predictions serve to optimize the functioning of the organism, thus promoting prediction regulation—a control-theoretic process that has been characterized in terms of allostasis (Sterling, 2012) or, more formally, as a prediction error (or free-energy) minimization (Seth et al., 2011; Seth, 2013; Suzuki et al., 2013).

Craig (2009) proposed that the interoceptive buffer may play a key role in time perception as well. This is because the buffer has a finite dimension and it can be easily filled up with interoceptive inputs, altering our perception of time. Specifically, high rate of salient stimuli can saturate the finite dimension of the buffer, speeding up the sampling frequency, effectively slowing (Tse et al., 2004; Campbell and Bryant, 2007; van Wassenhove et al., 2008; Wittmann and Paulus, 2008; Droit-Volet et al., 2011) the perception of time, which will appear to “stand still to the subjective observer” (Craig, 2009). Contrary, when the interoceptive buffer is not filled up “large intervals of time in the objective world can appear to pass quickly” (Craig, 2009).

Nonetheless, some studies revealed a paradox in time perception, when high rates of high salience stimuli can produce opposite effects (Droit-Volet and Meck, 2007; Droit-Volet and Gil, 2009). These findings may be explained by a functional processing lateralization of different interoceptive valences. Specifically, AIC appeared to be asymmetrically activated in definite conditions, whereas parasympathetic inputs are preferentially processed by the mid and left insula, while sympathetic activity is usually processed by the right AIC (Craig, 2009). According to Craig’s emotional asymmetry perspective, when we experience a dominant sympathetic arousal, stimuli processed in the right AIC speed up the sample rate frequency, accumulating pulses in the internal clock, leading therefore to overestimation of subjective time perception. Conversely, when we are engaged in a parasympathetic (e.g., affiliative) activation, stimuli are preferentially processed by the left AIC, leading to a subjective underestimation of time due to a lack of sympathetic activity (Craig, 2009).

The present study started from the hypothesis that time perception is intimately related to the functioning of the interoceptive buffer (Craig, 2009). However, while previous studies explored how bodily states can alter subjective time perception, our study focused on the opposite possibility: that is, the possibility to assess the degree of bodily input processing through distortions of interoceptive time perception. The key idea of Interoceptive Buffer saturation (IBs) index is to assess parasympathetic interoceptive stimuli, which are preferentially processed by the left insula (Gordon et al., 2013) for distortions in subjective time estimation due to the sympathetic workload of the coactive right insula. Parasympathetic interoceptive stimuli are reproducible through a specific kind of touch called affective touch (Olausson et al., 2002, 2016). This kind of touch encompasses activation of C-T afferents connected to the interoceptive system. To this goal, IBs design uses a custom developed “interoceptive stimulator” able to send interoceptive tactile inputs, consequently measuring distortion in subjective time estimation of these stimuli. The estimated degree of time distortion can therefore provide insight on subject’s bodily information processing due to the relative interference of other interoceptive inputs in the subjective time estimation.

To the best of our knowledge, interoceptive buffer has never been operationalized neither experimentally explored, therefore our results can provide powerful theoretical and clinical insights regarding the relationship between bodily states and interoceptive processing.

From a practical point of view, the rationale behind the IBs index is to reverse engineer the connection between the interoceptive matrix and the subjective perception of time, feeding interoceptive stimuli through the C-T afferents in the secondary touch system connected to the lamina I spinothalamocortical pathway. Using the IBs methodology, subjective overestimations of time would suggest a dominance of sympathetic arousal, while subjective underestimations of time would suggest the opposite condition.

According to the emotional asymmetry framework C-T (affective) touch (Olausson et al., 2002; Ackerley et al., 2014a,b; Liljencrantz and Olausson, 2014) comprises a parasympathetic activation primarily processed by the left AIC (Gordon et al., 2013) that might lead to an underestimation of time perception in healthy subjects (Craig, 2009). Nonetheless, contextual interferences caused by sympathetic stimuli—such as high arousing negative ones—processed by the right AIC should interfere with this endogenous time base creating distortions in perception towards an overestimation of time.

Numerous studies suggested an asymmetric effect of sympathetic and parasympathetic input on time perception. Indeed, time perception of parasympathetic interoceptive related stimuli appeared underestimated in normal conditions without sympathetic dominant activation (Ogden et al., 2015b); conversely, induced sympathetic stimuli are able to directly alter the internal time baseline, paradoxically leading to a more accurate perception (Mella et al., 2011) endorsing the notion of an emotional advantage for homeostatic regulation (Ogden et al., 2015a) as also confirmed by other authors (Angrilli et al., 1997; Buetti and Lleras, 2012; Droit-Volet et al., 2013; Pezzulo et al., 2018).

Comprehending and studying the interoceptive buffer has paramount value. Correct access to interoceptive information is key to allostasis and adaptive regulation of the organism, whereas different conditions such as anorexia nervosa (Pollatos et al., 2008) depression (Dunn et al., 2007) and chronic pain (Di Lernia et al., 2016b) appeared connected to alterations in interoceptive processing. Although several other indexes are currently available to assess different interoceptive factors (Garfinkel et al., 2015), IBs index may provide an advanced instrument with the ability not only to identify specific alterations but also the nature and the direction of the processes involved in these alterations. Common interoceptive deficits can be therefore connected to low buffer saturation levels indicating a diminished processing of stimuli arising from the body (i.e., anorexia nervosa, depression) but also with high saturation levels of negative arousal stimuli (i.e., chronic pain, anxiety) that can impair the perception of other inputs. Furthermore, IBs might also detect altered processing before the presence of actual deficits, providing an early indicator of clinical conditions not yet manifested.

To test IBs methodology, the study utilized a stream of interoceptive parasympathetic stimuli sent through the C-T afferents in the secondary touch system connected to the lamina I spinothalamocortical pathway, consequentially measuring for subjects’ distortions in time perception. Stimuli were delivered using an interoceptive stimulator specifically developed for the task. Considering aforementioned body of evidence, we hypothesized that healthy subjects will underestimate the duration of parasympathetic C-T interoceptive stimuli. Furthermore, we hypothesized that IBs index (i.e., the degree of duration estimation of interoceptive stimulation) will positively correlate with interoceptive accuracy (IAc) as a proxy of insula’s activity (Pollatos et al., 2007a). Moreover, we hypothesized that several psycho-physiological conditions that are known to interfere with AIC activity will influence IBs as well, leading to distortions in time perception accordingly to subjects’ sympathetic and parasympathetic balance. Specifically, we hypothesized that depressive symptoms (Dunn et al., 2007, 2010; Pollatos et al., 2009; Paulus and Stein, 2010; Wiebking et al., 2015) and eating disorder tendencies (Pollatos et al., 2008), will interfere in a negative direction, while anxiety symptoms (Whyman and Moos, 1967; Pollatos et al., 2009, 2014; Dunn et al., 2010; Paulus and Stein, 2010; Yoo and Lee, 2015) and other sympathetic stimuli (Ogden et al., 2015a) in a positive one.

We tested these hypotheses on healthy subjects assessing for common variables connected to interoceptive alterations. We assessed for risk of anorexia nervosa through EDI-3 (Garner et al., 1983) Drive for Thinness subscale (Eshkevari et al., 2012), and depressive symptoms through Beck Depression Inventory (BDI-II; Beck et al., 1961). Furthermore, we assessed anxiety factors through State Trait Anxiety Inventory (STAI; Spielberger et al., 1970) and endogenous interoceptive cardiac accuracy (IAc) as well (Schandry, 1981).

## Materials and Methods

### Participants

As part of a larger enlisting procedure in the university campus, 30 female subjects were recruited through consecutive sampling. Age (mean = 25.87 years, SD = 6.616) and BMI (mean = 20.827, SD = 2.24) were comparable to other healthy samples used in interoceptive studies (Garfinkel et al., 2015). Sample was composed only by female subjects to avoid somatosensory differences in perception due to gender related factors (Fillingim et al., 2009) as suggested by Ogden et al. (2015b). Furthermore, a solely female sample ensured no differences related to scales sensitivity, especially regarding eating disorder risk assessed by EDI-3 (Garner et al., 1983; Clausen et al., 2011; Eshkevari et al., 2012).

Exclusion criteria were the presence of current psychological or physical diagnoses, alterations in tactile perception (paraesthesia), allodynia and heart related conditions. Subjects were asked to avoid pharmacological medications in the 12 h before the experiment and nicotine and caffeine in the 2 h before the experiment.

This study was carried out in accordance with the recommendations of the Ethics Committee of Catholic University of Sacred Heart of Milan with written informed consent from all subjects. All subjects gave written informed consent in accordance with the Declaration of Helsinki (2008). The protocol was approved by the Ethics Committee of Catholic University of Sacred Heart of Milan.

### Procedure

On arrival, subjects received information about the experiment and proceeded to give written consent. Following a brief anamnestic interview and a series of psychological questionnaires, subjects were connected to a portable ECG device with Ag/AgCl electrodes to perform the IAc task. At the end of the task, electrodes were removed and subjects performed IBs, audio and video tasks. IBs, audio and video tasks were presented in randomized order.

### Psychological Assessment

After their arrival subjects took part to a brief anamnestic interview with a psychologist specialized in psychopathological and personality assessment. After anamnestic data collection they performed a battery of questionnaires. Depressive symptoms were assessed through the BDI-II (Beck et al., 1961). BDI-II is a 21 items self-report questionnaire with strong literature support. Anxiety symptoms were assessed through the well validated STAI (Spielberger et al., 1970). STAI is a 40-item scale that provided measure of state (STAI-S) and trait (STAI-T) anxiety. Risks for eating disorders were assessed through EDI-3 (Garner et al., 1983) risk subscales (Eshkevari et al., 2012). EDI-3 risk subscales assessed three different aspect of eating disorders risk: Drive for Thinness (DT), Bulimia (B) and Body Dissatisfaction (BD). Global risk index (EDRC) is composed summing the scores of these three subscales.

### Interoceptive Accuracy

An IAc score was established with the Schandry heart beat task (Schandry, 1981) through a portable ECG unit sampling at 250 Hz (Villarrubia et al., 2014a,b; Stojanović et al., 2015; Ševcík et al., 2015; Hugeng and Kurniawan, 2016) with Ag/AgCl electrodes. Time intervals were 25, 35, 45 and 100 s. Accuracy index was calculated with the following formula: 1/4∑(1 − (|recorded heartbeats − counted heartbeats|)/recorded heartbeats).

We focused on cardiac interoception not only because Schandry task is considered the standard measure for IAc, but also because Craig (2009) suggested that time perception might be fundamentally based upon cardiorespiratory function.

### Interoceptive Stimulation

**Figure 1:**
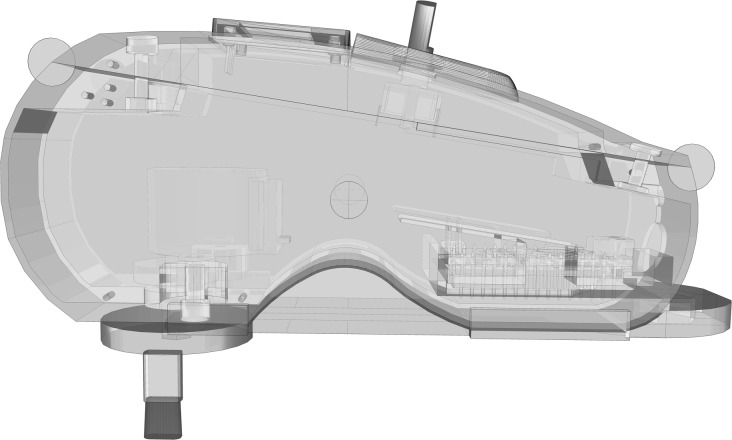
Interoceptive Stimulator.

The Interoceptive Buffer saturation (IBs) task applied interoceptive parasympathetic stimuli and asked the participants to estimate the duration of these stimuli. While there are several different kinds of interoceptive stimuli, for the goal of this study we used light touch as primary parasympathetic input (Ackerley et al., 2014b). Neuroanatomical evidence identified specific C tactile (C-T) afferents, which report directly to the AIC (Gordon et al., 2013) that are exquisitely sensitive to light touch (Vallbo et al., 1999; Ackerley et al., 2014b). Unmyelinated C-T fibers selectively respond to slow tactile brushing stimuli between 1 cm/s and 10 cm/s (Crucianelli et al., 2013; McGlone et al., 2014). Therefore, we used an instrument explicitly developed to distribute precise C-T stimuli (Figure 1) and specifically programmed for the IBs task.

Unmyelinated C-fiber tactile afferents are a specific type of fibers that can be found in not glabrous skin, constituting a secondary touch system that projects in the AIC (Olausson et al., 2002). They have been specifically identified in the facial skin (Nordin, 1990) and in the forearm (Vallbo et al., 1995, 1999; Wessberg et al., 2003). They are receptive to low force and low velocity strokes stimuli (Ackerley et al., 2014a,b). They exhibit a particular firing behavior with and inverted U relationship with stroke velocity (Löken et al., 2009), a tendency to fatigue (Iggo, 1960; Wessberg et al., 2003) and an after-discharge pattern that may present secondary characteristic such as delayed acceleration (Vallbo et al., 1995, 1999).

For such reason, the interoceptive stimulator has been designed to provide continuous interoceptive stimuli on the forearm, accounting for all C-T afferents factors. C-T afferents showed their maximal mean firing frequency at slow stroking velocity of 3 cm/s (Ackerley et al., 2014a), thus the interoceptive stimulator was set to move the brush tip at the exact speed of 3 cm/s (± 0.5 cm/s). The brush tip had an oval shape area of ≈35 mm^2^, to match the receptive field of a human C-T afferent (Wessberg et al., 2003; Liljencrantz and Olausson, 2014). The brush used a constant force <2.5 mN that is reported as C-T threshold by different authors (Nordin, 1990; Vallbo et al., 1999; Macefield, 2009), measured according to Vallbo et al. (1999). Furthermore, the brush design has been specifically developed to avoid fatigue and inexcitability (Iggo, 1960; Wessberg et al., 2003). As a matter of fact, C-T afferents decrease their firing rate to zero after 5 s of continuous stimulation (Liljencrantz and Olausson, 2014). Therefore, the brush tip moved in a circular pattern (10 cm of total length) at specific velocity of 3 cm/s plus a linear velocity of 0.5 cm/s. Considering circular pattern, receptive field area, brush tip dimensions (8.6 × 4.1 mm) and strokes velocity, a single cluster of C-T afferents received stimulation only for ≈0.3 s (0.28 s) within a single revolution, widely below the 5 s limit. The rest span between field stimuli allowed possible after-discharges to be expressed within the active test duration. Furthermore, 6 s resting phase between single trials and 1 min resting phase between stimulation blocks ensured an acceptable recovery of the C-T afferent fields. Delayed acceleration did not have a particular relevance for our design, considering that is not associated with subjective sensations (Vallbo et al., 1995, 1999).

### Interoceptive Buffer Saturation (IBs) Index

The IBs task is based on the Tactile Estimation Task (TET) for spatial recognition, but the protocol is adapted to temporal estimation (Kramer et al., 2011), thus providing a simple and non-invasive procedure to assess the degree of estimation of duration of the interoceptive stimulus. The task consists in delivering interoceptive brush strokes to subject’s left volar forearm, and successively asking for verbal time estimation (VET) of the time passed. The choice of VET^1^ is due to its effectiveness in probing time perception related to visual, auditory (Gil and Droit-Volet, 2011) and tactile dimensions (Ogden et al., 2015b) assessing for alterations in pulse rates within the pacemaker-accumulator (Mioni et al., 2016). Previous studies focused upon inducing distortions in the inner flow of time, trough external inductive stimuli. Conversely, IBs index will use the natural flow of time inside the interoceptive matrix to probe the saturation of interoceptive buffer.

Previous studies regarding interoceptive touch utilized stimulation at different velocity (>20 cm/s) or different target sites (i.e., palms of the hands) as control procedures. These control tasks rely upon A-fibers stimulation and these fibers are selectively activated by high velocities/high pressure stimuli or selectively present in the palm of the hands (Ackerley et al., 2014a,b; Liljencrantz and Olausson, 2014; McGlone et al., 2014). Nonetheless, these kind of control conditions were not suitable for the present study design due to the fact that perceived duration depends on perceived speed (Tomassini et al., 2011), excluding the possibility to use fast stimuli (speed > 20 cm/s) as control for interoceptive touch (Crucianelli et al., 2013; Ackerley et al., 2014a). Additionally, slow brush stimuli on the palm of the hands showed hedonic (parasympathetic) velocity-independent valence (Ackerley et al., 2014b), which could compromise IBs task design. Moreover, different clinical subjects exhibited deficits in tactile perception connected to A-fibers (Keizer et al., 2011, 2012; Stanton et al., 2013; Catley et al., 2014), thus A-fibers stimulation cannot be reliably used as control procedure in perspective of IBs index. Therefore, common audio and visual time estimation tasks were performed as control procedures upon separate sensory modalities.

Subjects were seated in a chair in a comfortable room while the clinician explained the experiment. They were instructed to give a “subjective time estimation of the stimulation”. They were instructed not to count the time passing, to pay attention to the feeling of the stimulation and to close their eyes for the entire duration of the procedure. To avoid counting, clinician suggested focusing on the physical sensation of the stimulation. Subjects were instructed that stimuli could last between “1 and 30 s approximately”, to avoid levelling underestimation effect (Ogden et al., 2015b). Subjects laid their left arm on the table in front of the clinician, bare skin (Tsakiris et al., 2011; Ackerley et al., 2014b). The clinician began the experiment starting the training block on the stimulator program delivering three brush stimuli of 7, 21, and 15 s, allowing the subject to familiarize with the procedure. No feedbacks were provided for effective durations or for performances. After each stimulus, a pause of 6 s allowed clinician to ask the subject, “*How many seconds do you feel the stimulus lasted*?” After the training procedure, clinician started the experimental task with randomized time durations. IBs index task delivered stimuli from 8 s to 18 s, at fixed intervals (8, 10, 12, 14, 16, 18). The experimental task provided six randomized predetermined stimuli per block, for three randomized blocks. Partial accuracy index for each time interval was calculated with the following formula: 1/3∑((time estimation − real time)/real time). Total index was calculated as mean of partial indexes.

### Audio and Video Time Estimation

Audio and video estimation tasks were common tasks frequently used and well described in the literature. Following Kramer et al. (2011), audio task presented a series of audio amplitude-steady complex tones. Video task replicated procedures from Wearden et al. (1998) substituting tones with a 4 × 4 cm light blue square presented on an iPad. Stimuli durations were 100, 200, 500, 1000 and 3000 ms replicated six times each and presented in random order. Audio task had a training procedure of 1000, 300 and 1500 ms. Video task had a training procedure of 1500, 1000 and 500 ms. Subjects were not informed about durations or performances (Wearden et al., 1998; Grondin, 2010; Kramer et al., 2011) but they were informed that stimuli could last between 50 ms and 4000 ms (Ogden et al., 2015b). After each stimulus subjects wrote the estimated duration in milliseconds on a data collection grid. Accuracy indexes were calculated with the following formula: 1/6∑((time estimation − real time)/real time). Total index was calculated as mean of partial indexes.

### Statistical Analyses

To verify underestimation of interoceptive C-T stimuli, a series of one sample *t*-test were used to determine if mean time estimation for every single interval differed significantly from real time values. The same procedure has also been applied to audio and video mean estimations.

A repeated measures ANOVA was run for IBs partial indexes between first, second and last stimulation block to verify that IBs task did not have any effect on the buffer.

A repeated measures ANOVA was run for IBs, audio and video accuracy indexes to identify significant differences between accuracy verbal estimation scores in different sensory modalities. Bonferroni *post hoc* was run to identify difference between groups.

Due to known limitations (Dunn et al., 2010) inherent assessment scales used, different factor structures have been implemented to better explore relationship between variables. Specifically, analyses implemented a two-factors structure for BDI-II to explore somatic and cognitive depression factors (Steer et al., 1999; Storch et al., 2004), and a four-factors structure for STAI to explore the presence and the absence of anxiety elements, both in state and trait dimensions (Vigneau and Cormier, 2008). Furthermore, scatterplot graphs and literature regarding interaction between interoception and depression (Dunn et al., 2007, 2010; Pollatos et al., 2009) suggested a quadratic negative relationship between BDI-II somatic factor and IBs.

Correlation analyses were run for variables of main interest. Moreover, a multiple regression analysis was conducted with IBs index as dependent variable and IAc, EDI DT subscale and BDI-II somatic symptoms as predictors.

Following literature suggestions (Pollatos et al., 2009; Dunn et al., 2010), a second multiple regression analysis was conducted with IBs index as dependent variable and IAc, EDI DT subscale, BDI-II somatic symptoms and interaction between the somatic component of BDI-II and the positive factor of STAI-S as predictors. All variables were centered before entering the regression analysis. The positive factor of STAI-S and the somatic factor of BDI-II were also z-standardized before calculating the interaction term. All the low level terms were left in the regression, as per methodological recommendations (Aiken et al., 1991). Residual plots were checked along with normality for observed standardized and unstandardized residuals. The same regression analyses were also conducted with audio and video accuracy indexes in substitution of IBs index.

## Results

### Sample Psychological Characteristics

Total sample of *N* = 30 showed high levels of trait anxiety (mean = 42.90, SD = 9.732) and moderate to high level of state anxiety (mean = 35.80 SD = 7.406). Results were comparable to other previous studies (Aktekin et al., 2001; Pollatos et al., 2007b, 2009).

Depressive symptoms were higher (mean = 7.60, SD = 5.157) than previous samples in interoceptive studies (Pollatos et al., 2009) with subjects in range of mild clinical depression (Steer et al., 1999; Storch et al., 2004) allowing us to meaningfully explore relationship with this variable.

**Figure 2:**
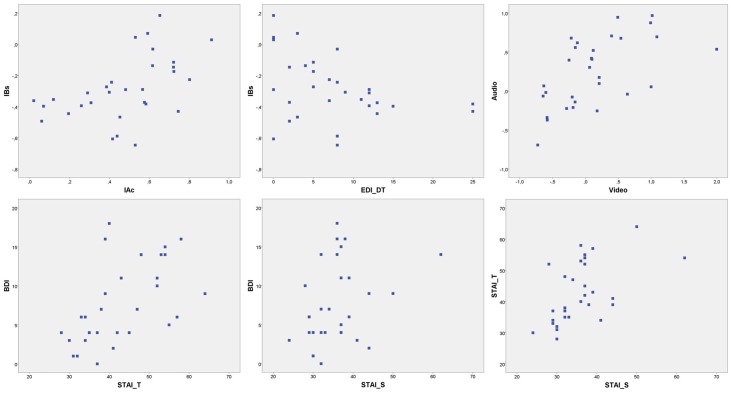
Scatterplot distributions. IBs, interoceptive buffer saturation index; IAc, interoceptive cardiac accuracy; EDI_DT, EDI drive for thinness risk subscale; Audio, Audio accuracy index; Video, video accuracy index; BDI, Beck Depression inventory; STAI_T, STAI trait anxiety; STAI_S, STAI state anxiety.

Nonetheless, depressive symptoms levels were comparable to normative data for similar populations (Storch et al., 2004). EDI-3 subscales assessed moderate risks of ED specifically related to BD (mean = 12.77, SD = 7.938; BD) and Drive for Thinness (mean = 7.47, SD = 6.564; DT). Several significant correlations were found between psychometric variables. Results are summarized in Tables 1, 2. Scatterplot distributions are provided in Figure 2.

**Table 1:** Sample characteristics and psychological assessment.

	*N*	Min	Max	Mean	SD
Age	30	21	47	25.87	6.616
BMI	30	17.263	28.548	20.827	2.240
BPM	30	51.32	107.89	74.80	11.85
IAc	30	0.020	0.912	0.473	0.231
IBs	30	−0.65	0.18	−0.29	0.20
Audio	30	−0.69	0.97	0.24	0.44
Video	30	−0.73	2.00	0.13	0.64
BDI	30	0	18	7.60	5.157
STAI Trait	30	28	64	42.90	9.732
STAI State	30	24	62	35.80	7.406
EDI_DT	30	0	25	7.47	6.564
EDI_B	30	0	11	2.70	2.793
EDI_BD	30	0	27	12.77	7.938
EDI_EDRC	30	2	58	22.93	13.831

*BMI, body mass index; BPM, heart beat per minute; IAc, interoceptive cardiac accuracy; IBs, interoceptive buffer saturation index; Audio, audio accuracy index; Video, video accuracy index; BDI, Beck Depression inventory; STAI, state and trait anxiety; EDI DT, drive for thinness; EDI B, bulimia; EDI BD, body dissatisfaction. EDI EDRC, composite risk index*.

**Table 2:** Correlation analyses for normally distributed variables of main interest.

Pearson’s	IAc	IBs	Audio	Video	BDI	STAI_T	STAI_S	EDI_DT	EDI_B	EDI_BD	EDI_EDRC
IAc	1										
IBs	0.504**	1									
Audio	0.160	0.098	1								
Video	0.259	0.270	0.588**	1							
BDI	0.045	−0.071	−0.146	0.197	1						
STAI_T	−0.029	0.091	−0.091	0.210	0.551**	1					
STAI_S^a^	−0.182	−0.155	−0.036	0.193	0.301	0.573**	1	0.307	0.214	0.437*	0.479**
EDI_DT	−0.175	−0.375*	0.185	0.056	0.019	0.257	-	1			
EDI_B^a^	−0.209	−0.171	−0.015	0.118	0.418*	0.497**	0.214	0.173	1	0.695**	0.635**
EDI_BD	−0.060	0.038	0.055	0.194	0.340	0.698**	-	0.383*	-	1	
EDI_EDRC	−0.147	−0.179	0.113	0.206	0.272	0.633**	-	0.743**	-	0.886**	1

**Correlation is significant at level 0.01 (two tails). *Correlation is significant at level 0.05 (two tails). ^a^Spearman’s correlation for not normally distributed variables. IAc, interoceptive cardiac accuracy; IBs, interoceptive buffer saturation index; Audio, Audio accuracy index; Video, video accuracy index; BDI, Beck Depression inventory; STAI, state and trait anxiety; EDI_DT, EDI drive for thinness risk subscale; EDI_B, EDI bulimia risk subscale; EDI_BD, EDI body dissatisfaction risk subscale; EDI_EDRC, EDI composite risk index.

### Interoceptive Accuracy

Interoceptive cardiac accuracy (Schandry, 1981) is a measure of heart-rate detection ability, and this sensitivity has been correlated with activation in the anterior insula (Pollatos et al., 2007a) and provided a standard measure of interoceptive awareness (Garfinkel et al., 2015). IAc mean score was 0.473 (SD = 0.231) and median was 0.50. IAc significantly positively correlated with IBs index (*r* = 0.504, *p* = 0.005). Results are summarized in Table 2.

### Interoceptive Buffer Saturation Index

**Figure 3:**
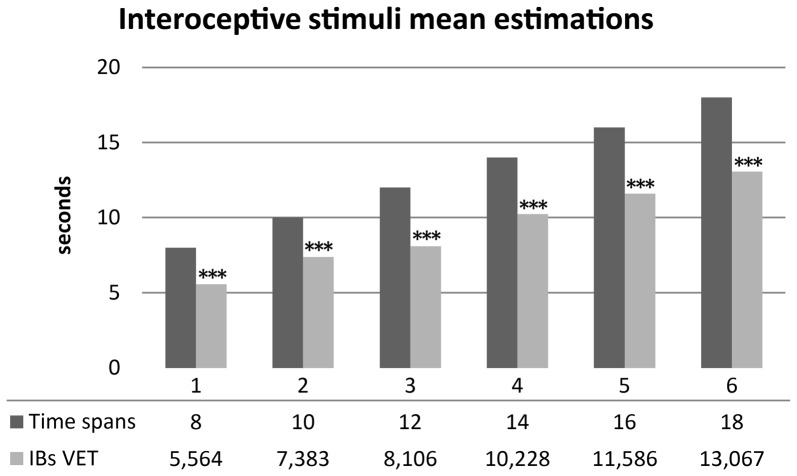
Mean time estimations of interoceptive tactile stimuli across different time spans. Interoceptive Buffer saturation (IBs) VET, mean verbal interoceptive time estimation. *p <0.05, **p < 0.01, ***p < 0.001.

As hypothesized, healthy subjects significantly underestimated durations of interoceptive stimuli. Mean scores were normally distributed. A series of one sample *t*-test showed significantly underestimation of mean time perception of interoceptive stimuli for all time spans: 8 s (*t*_(29)_ = −7.396, *p* < 0.001, *d* = −1.35), 10 s (*t*_(29)_ = −5.628, *p* < 0.001, *d* = −1.02), 12 s (*t*_(29)_ = −8.162, *p* < 0.001, *d* = −1.49), 14 s (*t*_(29)_ = −7.004, *p* < 0.001, *d* = −1.27), 16 s (*t*_(29)_ = −6.955, *p* < 0.001, *d* = −1.27), 18 s (*t*_(29)_ = −7.111, *p* < 0.001, *d* = −1.298). Results are summarized in Figure 3.

As hypothesized, IBs task did not have any manipulative effect on the buffer, confirming the effectiveness of the task as assessment instrument. A repeated measures ANOVA showed no statistically significant differences on partial IBs indexes between first, second and last stimulation block (*F*_(2,58)_ = 0.142, *p* = 0.868, $\eta_{\text{p}}^{2}$ = 0.005). Results indicated that IBs task did not alter subjects’ endogenous baseline^2^.

As hypothesized, Pearson’s correlation analyses showed a significantly positive linear relationship between IBs and IAc (*r* = 0.504, *p* = 0.005) and significantly negative linear relationship between IBs and EDI Drive for Thinness (*r* = −0.375, *p* = 0.041). Results are summarized in Table 2.

Scatterplot and literature (Dunn et al., 2007, 2010) suggested a quadratic relationship between depression and interoceptive processing. As hypothesized, IBs correlation with a quadratic term of BDI-II somatic factor approach significance for a negative relationship (*r* = −0.358, *p* = 0.052).

A multiple regression analysis was conducted with IBs index as dependent variable and IAc, EDI Drive for Thinness and quadratic transformation of BDI-II somatic factor as predictors. Sample dimension assured an adequate power (Harrell, 2015) for the analysis. As hypothesized, variables significantly predicted IBs (*R*^2^ = 0.443, *F*_(3,26)_ = 6.880, *p* < 0.001). Beta standardized coefficients indicated that IAc significantly positively predicted IBs (*β* = 0.420, *p* = 0.009) while EDI Drive for Thinness (*β* = −0.306, *p* ≤ 0.05) and somatic depressive factor of BDI-II (*β* = −0.324, *p* ≤ 0.05)^3^ significantly negatively predicted saturation levels in the interoceptive buffer.

To explore relationship between anxiety and interoceptive buffer levels, a second multiple regression analysis was conducted with IBs index as dependent variable and IAc, EDI Drive for Thinness, BDI-II somatic quadratic transformation and interaction between the somatic component of BDI-II and the positive STAI-S factor as predictors (Pollatos et al., 2009; Dunn et al., 2010; Paulus and Stein, 2010). Variables significantly predicted IBs (*R*^2^ = 0.529, *adjusted* *R*^2^ = 0.406, *F*_(6,23)_ = 4.306, *p* < 0.005). IAc positively predicted saturation levels in the buffer (*β* = 0.465, *p* = 0.005) while EDI Drive for Thinness (*β* = −0.345, *p* = 0.028) and BDI-II somatic factor (*β* = −0.394, *p* = 0.018) significantly negatively predicted IBs. Interaction term between somatic BDI-II factor and positive STAI-S factor approached significance (*β* = 0.318, *p* = 0.083). Sample size was slightly underpowered for this regression model, nonetheless methodological recommendations indicated that multiple regression is quite robust to small sample if *adjusted* *R*^2^ is considered in substitution of *R*^2^ (Austin and Steyerberg, 2015).

### Audio and Video Time Estimation

**Figure 4:**
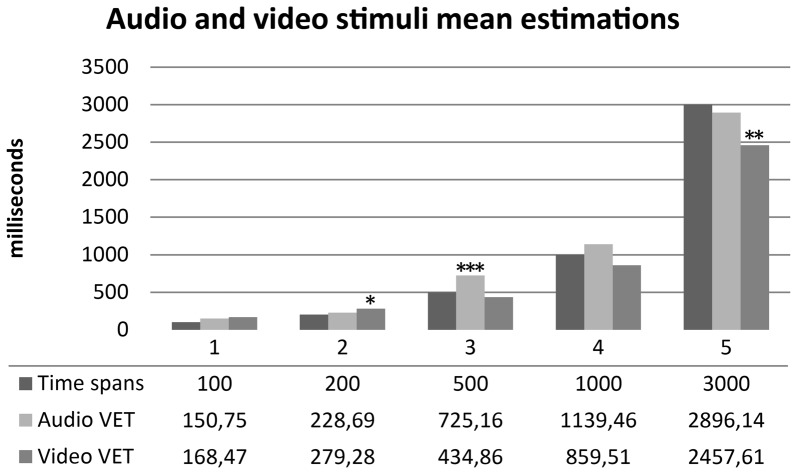
Mean time estimation of audio and video stimuli across different time spans. Audio VET, mean verbal audio time estimation; Video VET, mean verbal video time estimation. *p <0.05, **p < 0.01, ***p < 0.001.

One sample *t*-tests were performed between audio and video time estimations and real time spans. Wilcoxon tests were performed for not-normally distributed variables. Audio estimations did not show any statistically significant difference for 100, 200, 1000 and 3000 ms time spans (*p* > 0.05) compared to real time spans. Subjects significantly overestimated 500 ms audio stimuli (*t*_(29)_ = 3.958, *p* < 0.001, *d* = 0.722). Video estimations did not show any statistically significant difference for 100, 500, 1000 ms time spans (*p* > 0.05) compared to real time spans. Subjects significantly overestimated 200 ms video stimuli (*t*_(29)_ = 2.242, *p* = 0.033, *d* = 0.409) and significantly underestimated 3000 ms video stimuli (*t*_(29)_ = −3.032, *p* = 0.005, *d* = −0.553). Results are summarized in Figure 4. One sample *t*-tests for paired sample were performed between normally distributed audio and video time estimations. Wilcoxon tests were performed for not-normally distributed values. Subjects judged audio stimuli significantly longer than video stimuli in time spans 500, 1000 and 3000 ms (*p* = 0.005). A *t*-test for paired sample was performed between audio and video accuracy indexes without any statistically significant difference (*p* = 0.232). Audio and video accuracy indexes significantly positively correlated (*r* = 0.588, *p* = 0.001). Results are summarized in Table 2. Regression analyses for audio (*p* = 0.490) and video (*p* = 0.478) accuracy indexes were performed with IAc, EDI_DT and BDI-II somatic factor. Both models failed to reach statistical significance^4^.

### Relationship Between Measures

A repeated measures ANOVA with a Greenhouse-Geisser correction was performed between IBs, audio and video accuracy indexes (factor accuracy). Results reported a statistically significant difference between accuracy indexes (*F*_(1.806,52.371)_ = 16.037, *p* < 0.001, $\eta_{\text{p}}^{2}$ = 0.356). *Post hoc* tests using Bonferroni correction revealed that IBs accuracy index was significantly underestimated compared to both audio (*p* < 0.001) and video (*p* = 0.003) accuracy indexes. No statistically significant differences were found between audio and video accuracy indexes (*p* = 0.697) although audio stimuli (mean = 0.241, SD = 0.080) were judged longer than video ones (mean = 0.126, SD = 0.116).

Several statistically significant correlations were found between psychometric variables. Results are summarized in Table 2.

## Discussion

The study explored the connection between time perception and interoceptive processing, proposing the IBs index as a novel construct able to provide information about co-active processing within the interoceptive matrix (AIC; Craig, 2002, 2003, 2009). The IBs index relied upon a verbal estimation task of interoceptive stimuli sent through the secondary touch system connected to the lamina I spinothalamocortical pathway. Bidirectional distortions in the subjective time perception of these stimuli provide information about sympathetic and parasympathetic activity in the cortex. The interoceptive buffer is a key component of the interoceptive matrix and it constantly processes and compares information arising from the body, creating bodily meta-representations shaped upon the asymmetrical relationship between the right and the left insula. Understanding saturation levels within the buffer can provide meaningful insight about this relationship, whereas high levels of saturation may indicate large amount of sympathetic stimuli processed in the right insula, while opposite results may indicate that the interoceptive system is actually processing a low amount of high salience sympathetic stimuli from the body.

Recent evidence identified deficits in the insula connected with radically different psychopathological conditions. Chronic pain (Schaefer et al., 2012; Weiss et al., 2014; Duschek et al., 2015), depression (Dunn et al., 2007, 2010; Pollatos et al., 2009; Sprengelmeyer et al., 2011; Sliz and Hayley, 2012; Stratmann et al., 2014), eating disorders (Pollatos et al., 2008; Fischer et al., 2016) and several other conditions (Rosso et al., 2010; Wylie and Tregellas, 2010; Hatton et al., 2012; Naqvi et al., 2014) comprised alterations within the interoceptive network. Nonetheless, it is not clearly understood how radically different conditions can impair the insula network in a similar way.

In our previous work (Di Lernia et al., 2016a), we suggested that IBs may provide a better measure of interoceptive processing compared to other available indexes, because it permits to disentangle the differential effects of interoceptive alterations within the matrix. Conceptualizing these alterations in terms of buffer saturation levels suggests that high levels of negative arousing stimuli may impair interoceptive processing through widespread interferences (i.e., interoceptive noise), whereas low stimuli processing may reduce body awareness (i.e., interoceptive silence), with both conditions leading to deficits in interoceptive perception.

The design of the study did not include non-interoceptive control stimuli, as explained in the “Materials and Methods” section. This was due to the nature of the task we adopted, in which different stimulation speeds—which are necessary to perform non interoceptive stimulation—would have yielded incomparable results in a time estimation task. A second equally important implication that defined IBs design is also that interoceptive tactile stimuli are never exclusively related to C-T fibers. Neurophysiological evidence indicated that every C-T related stimulus always comprises a concomitant activation of myelinated tactile fibers processed by the somatosensory cortex (Roudaut et al., 2012). Although a mechanical stimulation such as the one delivered by the interoceptive stimulator can be tuned to produce massive peak activations in C-T tactile afferents, every interoceptive tactile stimulus always partially activates other myelinated fibers (Crucianelli et al., 2013; Ackerley et al., 2014a,b). Therefore a non-interoceptive control task would have yielded incomparable, albeit informative, results from IBs perspective. This was, among others, one of the prominent reasons according to which we have chosen different sensory modalities (audio and video) as control procedures; also in agreement with protocols to study emotions and time perception that usually compared results across different unrelated perceptive systems. Nonetheless, considering IBs design, only correlations between the index with the Schandry’s task and the questionnaires can be used for an interpretation regarding interoceptive processes.

As hypothesized, results confirmed that healthy subjects significantly underestimated the duration of parasympathetic interoceptive C-T stimuli, compared to actual time spans. Results confirmed previous findings about C-T processing (Leonard et al., 2014; Ogden et al., 2015b), supporting the methodology used in the present study and the effectiveness of the device developed. Comparing our results with previous studies about sympathetic stimuli (Droit-Volet et al., 2011; Ogden et al., 2015a) also endorsed Craig’s emotional time asymmetry framework and consequently the rationale behind the IBs index. Significant underestimation of C-T stimuli suggested that healthy subjects without dominant sympathetic activation tend to have a reduced stimuli processing in the right insula buffer, matching a low pulse rate within the interoceptive internal clock therefore experiencing a contraction in time perception due to a lack of salience in bodily arousal.

As hypothesized, IBs index positively correlated and was positively predicted by interoceptive cardiac accuracy (IAc). This result provided strong support regarding the validity of IBs index due to the ontological connection between the buffer and the degree of activation of the insula (Pollatos et al., 2007a). Nonetheless, correlation was sufficiently distinct to suggest that IBs index and interoceptive cardiac accuracy are two different, albeit intertwined, constructs.

IBs index negatively correlated and was negatively predicted by EDI-3 Drive for Thinness subscale, suggesting an interesting insight regarding the connection of the buffer with body perception. IAc is significantly reduced in clinical subjects with anorexia nervosa (Pollatos et al., 2008); nonetheless, the nature of these deficits is not really understood even though they can be equally found in radically different conditions (Di Lernia et al., 2016b). In our sample, we found no significant correlation between IAc and Drive for Thinness, as expected for healthy females that did have neither interoceptive deficits nor anorexia nervosa. Conversely, we found a negative correlation between IBs and Drive for Thinness risk, suggesting that predisposition for anorexia nervosa can be connected to low saturation levels within interoceptive buffer. Considering our results, low saturation levels in the buffer (IBs) can be described as a hypo-saturation condition whereas the interoceptive matrix processes lower amounts of high salience (i.e., hunger, thirst etc.) stimuli from the body. This impaired processing activity can therefore lead to pervasive alterations in body perception and, ultimately, to the interoceptive deficits and bodily distortions identified in anorexia nervosa (Pollatos et al., 2008). Recent fMRI evidence showed consistent alterations in the insula network both on functional (Gaudio et al., 2015; Kerr et al., 2016) and structural level (Gaudio et al., 2017) indicating impaired processing of high salience bodily stimuli in anorexia nervosa (Wierenga et al., 2015), therefore supporting our results. Moreover, anorexic subjects showed no impairment in a time duration task connected to non-interoceptive neutral tactile stimuli (Spitoni et al., 2015) fostering the conclusion that IBs results are strictly connected to processing within insula network and its relationship with body perception.

Depression and anxiety symptoms did not show a direct linear correlation neither with IBs nor IAc accuracy indexes, partially diverging from previous literature results (Pollatos et al., 2009). This difference could be due either to limited sample dimension in our study or to the lack of sensitivity of the two assessment scales we used, which would be in keeping with other previous results (Dunn et al., 2010). Our results indicate a poor effectiveness of BDI-II and STAI questionnaires to assess interoceptive dimensions of depression and anxiety. Dunn et al. (2010) suggested that BDI-II and STAI questionnaires may have confounding overlapping constructs, more directly connected to global severity measures and therefore they might be relatively inappropriate to measure interoceptive components. Our findings, related to a small albeit representative sample, confirmed this perspective also through a significant correlation between the scales.

To effectively assess bodily components of depression and anxiety, we therefore implemented several well validated factor structures. BDI-II was divided into two-factors connected to somatic and cognitive components of depression (Steer et al., 1999; Storch et al., 2004). STAI was divided into four-factors that described positive and negative items of anxiety, both in state and trait conditions (Vigneau and Cormier, 2008).

Consequently, we focused on the somatic factor of BDI-II and a more accurate analysis identified a not-linear negative relationship (Dunn et al., 2007) between depressive somatic symptoms and IBs levels. Our results suggested that depression negatively interfered with saturation levels in the buffer promoting a hypo-saturation condition in which depressive somatic components appeared to be related to a diminished processing of high valence arousing stimuli in the right insula. These results are also supported by previous neurophysiological evidence that indicated a hypo-response (Wiebking et al., 2015) along with reduction in gray matter volume of the insula (Sprengelmeyer et al., 2011; Stratmann et al., 2014) in major depressive disorders.

In our study, anxiety measured through STAI questionnaire failed to provide statistically significant results. This could be due to the small sample size but more probably to the general construct within the scale that is primarily oriented to global severity measures rather than anxiety positive arousal symptoms (Dunn et al., 2010). Following literature recommendations (Pollatos et al., 2009; Dunn et al., 2010; Paulus and Stein, 2010), we nonetheless explored the interaction between somatic depressive factor and positive state anxiety factor. The interaction approached a positive statistical significance suggesting some further considerations that, nonetheless, must be considered with caution. Specifically, comorbidity between somatic depressive and anxiety symptoms appeared to have a moderately positive influence upon saturation levels in the buffer, suggesting a biological prevalence of sympathetic arousal preferentially processed in the right insula. Several authors backed this perspective that relied upon the role of high arousal stimuli in the optimal functioning of the organism (Jänig, 2008; Wiech et al., 2010; Uddin, 2015) supporting the notion of an emotional advantage in perception processing for homeostatic regulatory purposes, which foster enhanced attention to bodily signals (Ogden et al., 2015a; Yoo and Lee, 2015).

Interestingly, healthy subjects did not systematically underestimate durations of audio and video stimuli. Moreover, a significant difference between IBs, audio and video accuracy indexes suggested secondary implications regarding time perception theories. Specifically, our findings indicated different pacemaker-accumulator units, connected to different sensory modalities. Audio and video indexes showed a positive significant correlation between each other, but failed to significantly correlate to interoceptive time perception indicating separated time processing pathways. Moreover, regression analysis also failed to be significant for the audio and video accuracy indexes, suggesting that audio and video time estimation probably referred to different processing circuits, partially distinct from the interoceptive system. These results are supported by recent literature (Mioni et al., 2016) according to which time perception in depression showed inconclusive results (Thones and Oberfeld, 2015) along with not-inductive time studies in anxiety that also provided mixed results (Lueck, 2007; Brown, 2016).

A possible explanation for the significant difference between audio, video and IBs accuracy indexes can be related to the different time spans used, suggesting that time estimation in the range of milliseconds is not susceptible to the same psycho-physiological components that contribute to IBs index. Nonetheless, different studies used time spans within milliseconds range to identify a parasympathetic (Ogden et al., 2015b) and sympathetic (Droit-Volet and Meck, 2007; Gil and Droit-Volet, 2011, 2012; Droit-Volet et al., 2013; Ogden et al., 2015a; Thones and Oberfeld, 2015; Yoo and Lee, 2015; Mioni et al., 2016) influence upon the internal clock. Moreover, time spans in the range from seconds to subseconds have often been considered connected to the same time perception mechanisms (Church, 1984) whereas evidence from literature found no difference in time intervals as a factor (Macar et al., 2002). Furthermore, evidence from computational neuroscience suggested that time estimations from millisecond to seconds rely upon the same encoding processing patterns (Karmarkar and Buonomano, 2007). Karmarkar and Buonomano (2007) also suggested that short time intervals—as the lower ones in the audio and video estimation tasks—are also probably connected to a nonlinear time metric encoded in local neural networks and therefore they should be even more sensible to the psychophysiological variables that contribute to IBs index. In a similar manner, Craig suggested that the interoceptive network might “provide a basis for the human capacity to perceive and estimate time intervals in the range of seconds to subseconds” (Craig, 2009) also supporting the possibility to coherently explore different time spans. This evidence suggested that time spans in milliseconds range should be sensible to alterations in bodily processing, thus excluding the duration of the stimuli as a factor and allowing the design of the study to select the most appropriate and the most sensible time intervals for each sensory modality.

Considering our results, it is therefore possible that time perception is processed differently depending on the sensory modality investigated and that time information collected by several different internal clocks are sub-sequentially merged in a global time perception awareness, composed by different elements according to their contextual salience. This hypothesis remains to be tested in future studies.

### Interoceptive Buffer, Active Inference and Predictive Coding

From a more formal, predictive coding (or interoceptive inference) perspective, buffer saturation can be understood in terms of well-known mechanisms of precision (or gain) control during (Bayesian) inference. One essential role of interoceptive inference is integrating various interoceptive signals to form an estimate of the state of the body (e.g., heart rate as well as gastric and respiratory signals to assess momentary fatigue or fear), based on which the organism can take adaptive action (Pezzulo, 2014; Pezzulo et al., 2015). Importantly, while forming this estimate, the reliability of all the interoceptive signals (as well as of prior information) must be evaluated, too; signals that have higher salience or precision (or lower uncertainty) are weighted more and have higher impact on the inference, whereas lower-precision signals have lesser impact and in some cases can be also disattended or ignored—thus implementing a form of precision (or gain) control over interoceptive processing. Within this framework, signals that are highly salient or have high behavioral significance (e.g., threats) would be plausibly afforded a high gain, hence dominating the inference and reducing the impact of other signals (aka, saturating the buffer). This is in general an adaptive mechanism, which would afford priority to the most important signals; but in some cases, it can become maladaptive. For example, expecting the environment to be too volatile might lead to over-sampling it (like, e.g., in anxiety) and to a systematic overestimation of the precision of sensory and interoceptive signals. Conversely, other pathological conditions (e.g., anorexia) may be linked to interoceptive precision down-regulation, and thus to a systematically diminished processing of stimuli arising from the body (aka, silencing one of the channels of the buffer rather than saturating it). This framework would shed light on the different ways interoceptive inference can become aberrant, due (for example) to the fallacious up- or down-regulation of the precision of interoceptive (or other) signals—and how this in turn would produce maladaptive behavior that is specific of different clinical conditions (Friston et al., 2014).

### Clinical Implication of Interoceptive Buffer Saturation

IBs index can provide insight regarding interoceptive alterations in apparently different conditions, nonetheless this knowledge may serve other purposes besides simple assessment. Specifically, pathological conditions with an hyper-saturated status might present alterations in bodily signals connected to an overflow of sympathetic-related stimuli in the buffer (i.e., interoceptive noise) lowering the access to other interoceptive information (Di Lernia et al., 2016a). Conversely, hypo-saturated conditions may be connected to a general impairment of whole stimuli processing that limited inputs (i.e., interoceptive silence) from actually reaching AIC.

If confirmed, this perspective may have important implications beyond assessment, foreseeing the concept of “interoceptive treatments”. As a matter of fact, clinical conditions (i.e., anxiety or chronic pain) characterized by an overflow of sympathetic related interoceptive stimuli (i.e., hyper-saturation) may benefit from an interoceptive parasympathetic stimulation with low variance to promote co-activation of the left insula, reducing processing in the right AIC with the ultimate goal of contrasting symptoms severity. Conversely, if a clinical condition will show hypo-saturation due to a block that impairs interoceptive information to actually reach the insula (i.e., depression or anorexia nervosa), interoceptive treatments based upon interoceptive stimuli with high variance could provide a way to foster high salience processing in the right AIC, restoring a correct access to bodily information. If confirmed, albeit highly theoretical, these perspectives can provide innovative insights in treatment fields, for several different psychopathological conditions.

### Limitations

Several limitations impaired study design and results. Sample size was reduced, nonetheless consecutive sampling provided highly informative data, and literature confirmed an adequate statistical power (Austin and Steyerberg, 2015; Harrell, 2015) related to number of subjects and statistical analyses performed.

A solely female sample ensured comparable results on somatosensory related tests (Fillingim et al., 2009) and psychological assessment (Garner et al., 1983; Clausen et al., 2011; Eshkevari et al., 2012) nevertheless the design of the study did not explore IBs in male population.

The study utilized audio and video stimuli as control procedures due to the fact that somatic non-interoceptive conditions (i.e., different velocities or different body locations) were not suitable within IBs design. This choice allowed us to explore time perception upon different sensory modalities. However, the absence of somatic control stimuli did not allow us to explore possible differences in somatic non-interoceptive processing. Moreover, interoceptive stimulation always presents concomitant activation of Aβ receptors (Ackerley et al., 2014a,b; Ogden et al., 2015b) therefore non-interoceptive stimuli with a parasympathetic component may correlate with IBs index. These problems might be addressed by using reliable somatic controls, such as vibrational stimuli that specifically target (Liljencrantz and Olausson, 2014) Pacinian corpuscles at a frequency between 150 Hz and –300 Hz (Roudaut et al., 2012). Nonetheless, this kind of stimulation requires a specifically designed prototype not available during this study.

BDI-II and STAI questionnaires presented several limitations regarding construct measures, overlapping theoretical relevance, and effectiveness to measure interoceptive components (Dunn et al., 2010). In our study, these limitations were partially addressed through several factor structures nonetheless a different model of anxiety and depression could provide better assessment of interoceptive dimensions. Specifically, the tripartite model of mood disorders (Clark and Watson, 1991) might provide a more effective framework for interoceptive related components. This model relies upon the Mood and Anxiety Symptom Questionnaire (MASQ-S; Watson et al., 1995; Keogh and Reidy, 2000) to assess several dimensions connected to symptoms of nonspecific general distress, symptoms specific to depression and symptoms specific to anxiety. Specifically, MASQ-S subscales Anxious Arousal (17 items) and Anhedonic Depression (22 items) might provide better results compared to global severity measures reported by BDI-II and STAI. Unfortunately, Italian validation of the instrument was not available at the time of this study.

Lastly, literature suggested that belief and expectations can limit Schandry task validity to assess for interoceptive cardiac awareness (Ring et al., 2015). Nonetheless, heart beat perception task (Schandry, 1981) remained the most validate and most reliable instrument for IAc assessment (Pollatos et al., 2007a; Critchley and Garfinkel, 2015; Garfinkel et al., 2015).

### Conclusion and Future Directions

Our results provided a powerful theoretical insight regarding the relationship between time perception, interoceptive processing and psycho-physiological conditions. Distortions in interoceptive time perception recorded by IBs index appeared to be function of different sympathetic and parasympathetic co-activation processes within insula cortex.

Nonetheless, different questions remain to be explored, promoting future research directions. Specifically, it will be fundamental to test IBs index proposal on different pathological conditions connected to insula deficits, such as chronic pain, anorexia nervosa and depression also comparing results to healthy subjects’ performances.

Furthermore, IBs results may support the concept of “interoceptive treatments” as clinical applications for a new non-pharmacological option to treat a variety of disorders characterized by interoceptive network alterations. These kinds of treatments, tailored upon specific dysregulations of the insula cortex, might provide powerful instruments to reduce symptoms severity in those conditions that are resistant to pharmacological medications, without any side effect or interaction with concomitant therapies (Di Lernia et al., 2016a; Riva et al., 2016, 2017). Interoceptive treatments may therefore prove themselves an effective option to promote a balanced functioning of the organism both in clinical and healthy population, opening a brand new field of medicine and neuroscience.

## Author Contributions

DDL: conceptualization, investigation, writing—original draft, hardware and software development. DDL and SS: methodology. SS, GP, EP, PC and GR: writing—review, editing and supervision.

## Conflict of Interest Statement

The authors declare that the research was conducted in the absence of any commercial or financial relationships that could be construed as a potential conflict of interest.

## Footnotes

^1^On the other hand, we dismissed time production and reproduction tasks because that are usually dependent upon attention and working memory processes “rather than variation in internal clock” (Mioni et al., 2016).

^2^IBs is proposed as an assessing instrument; therefore we checked if the stimulation had any kind of effect upon subject’s temporal perception. We verified this by confronting mean estimations of the first stimulation block with mean estimations on the last stimulation block. An effect of the interoceptive stimulation would have produced variations in temporal estimation across blocks. Since we did not observe significative differences in time estimation between first and last stimuli, across all the protocol, we concluded that the stimulation itself did not have any impact on subject’s endogenous time perception.

^3^BDI-II somatic factor quadratic transformation (*β* = −0.324, *p* = 0.037) | Model with untransformed term *R*^2^ = 0.461, *F*_(4,25)_ = 5.337, *p* < 0.003, IAc *β* = 0.420, *p* = 0.01, EDI_DT *β* = −0.308, *p* = 0.05, BDI-II somatic untransformed term *β* = −0.136, *p* = 0.369, BDI-II somatic quadratic transformed term *β* = −0.303, *p* = 0.053. Residuals K-S > 0.200, S-W = 0.928.

^4^Model with untransformed term: Audio accuracy index *p* = 0.611, Video accuracy index *p* = 0.288.
